# Incomplete quality of life data in lung transplant research: comparing cross sectional, repeated measures ANOVA, and multi-level analysis

**DOI:** 10.1186/1465-9921-6-101

**Published:** 2005-09-08

**Authors:** Karin M Vermeulen, Wendy J Post, Mark M Span, Wim van der Bij, Gerard H Koëter, Elisabeth M TenVergert

**Affiliations:** 1 Office for Medical Technology Assessment, University Medical Center Groningen, the Netherlands; 2 Department of Pulmonary Diseases, University Medical Center Groningen, the Netherlands

## Abstract

**Background:**

In longitudinal studies on Health Related Quality of Life (HRQL) it frequently occurs that patients have one or more missing forms, which may cause bias, and reduce the sample size. Aims of the present study were to address the problem of missing data in the field of lung transplantation (LgTX) and HRQL, to compare results obtained with different methods of analysis, and to show the value of each type of statistical method used to summarize data.

**Methods:**

Results from cross-sectional analysis, repeated measures on complete cases (ANOVA), and a multi-level analysis were compared. The scores on the dimension 'energy' of the Nottingham Health Profile (NHP) after transplantation were used to illustrate the differences between methods.

**Results:**

Compared to repeated measures ANOVA, the cross-sectional and multi-level analysis included more patients, and allowed for a longer period of follow-up. In contrast to the cross sectional analyses, in the complete case analysis, and the multi-level analysis, the correlation between different time points was taken into account. Patterns over time of the three methods were comparable. In general, results from repeated measures ANOVA showed the most favorable energy scores, and results from the multi-level analysis the least favorable. Due to the separate subgroups per time point in the cross-sectional analysis, and the relatively small number of patients in the repeated measures ANOVA, inclusion of predictors was only possible in the multi-level analysis.

**Conclusion:**

Results obtained with the various methods of analysis differed, indicating some reduction of bias took place. Multi-level analysis is a useful approach to study changes over time in a data set where missing data, to reduce bias, make efficient use of available data, and to include predictors, in studies concerning the effects of LgTX on HRQL.

## Background

Lung transplantation has become an accepted treatment option for appropriately selected patients with end-stage lung disease. Besides clinical outcome measures such as survival, Health Related Quality of Life (HRQL) has become an increasingly important endpoint in studies regarding the effectiveness of lung transplantation. Studies in which HRQL was included as an outcome measure generally report improvements across many domains of HRQL after lung transplantation [1-7]. The aim of the present study was twofold. First, to address the problem of missing data in the field of HRQL and lung transplantation, and secondly to compare results from different methods of analysis in a data-set where missing data occur in order to show the value of each type of statistical method used to summarize data.

In many studies, HRQL is assessed longitudinally by means of questionnaires, which are presented to the patients at several predetermined time points in order to evaluate changes over time. Unfortunately, missing assessments are frequently encountered and can be caused by a variety of factors. A possible cause for missingness of data can be poor data management, for example when a research employee 'forgets' to hand out a questionnaire to a patient (logistic reason). When the burden on the patient is too high, for example due to a large number of questionnaires, or question difficulty this can also be a reason for dropping out (methodological reason). In the examples mentioned above, it is unlikely that the reason for missing is related to the patients health status. Other reasons for missingness are health problems or side effects of therapy due to which patients are temporarily unable to complete the questionnaire. An other example of a reason for missingness is the death of a patient. In these cases the missingness is reflects the patients health status. Missingness of data due to logistic or methodological reasons, can be prevented. Consequently, in this case the best way to handle the missing data problem is prevention. Missingness of data caused by patient related factors is more unpreventable.

The missingness of data has two major undesirable effects. First, if missingness is correlated with the outcome one is interested in, ignoring it will bias the results. For example, when missingness is caused by serious health problems, patients with missing assessments will differ on health status from patients who have completed all forms. Consequently, results of patients with complete forms cannot be generalized to the entire population: conclusions are only applicable to the group of 'completers' who have better health status than other patients in the population. A second complication associated with missing is the loss of efficiency. Because most statistical software packages automatically drop subjects with one or more missing assessments, it causes loss of efficiency due to reduced sample sizes in the analysis. Few researchers in the field of lung transplantation have acknowledged the problem of missing HRQL data [1,8]. However, no consensus could be found in the LgTX literature about the appropriate statistical method for dealing with it. Moreover, the choice for a particular statistical method strongly depends on the study objective under investigation.

Irrespective of the reasons for, and the magnitude of the missing data problem, two methods of analyzing data are commonly performed in studies regarding the effects of lung transplantation on HRQL. First, especially in the earlier years when the number of transplanted patients was still relatively small, cross sectional analyses were usually performed. In this type of analyses, at two or more time points, all available data at that specific point are analyzed. These kind of analyses result in conclusions for different groups of patients at the various time points. Thus, in cross sectional analyses, the longitudinal character of the data set is ignored. When the research aim is to assess changes over time, cross sectional analyses are not suitable. However, this method is acceptable for descriptive purposes and has the advantage that it makes efficient use of the available data at each time point.

When studying changes over time, longitudinal analyses are preferred [9]. However, when repeated measures techniques are used, most commonly used software packages exclude the entire patient with one or more missing assessments from the analysis. Consequently, only patients who have completed all questionnaires (complete cases) are included. When the research is aimed at describing a specific subgroup of, for example surviving patients, complete case analysis may be appropriate. In addition, complete case, but also cross sectional methods can be used in case missing forms are completely randomly distributed, and the reduced data represent a randomly drawn sub-sample of the original data-set [10]. However, when patients with incomplete data differ from patients with complete data, and missingness can be predicted from other observed variables, complete case analysis may not be valid. In that case, an alternative method of analysis has to be used to assess changes over time. In our study, the methods we will focus on are likelihood based, which provide estimates based on all available data. These methods have been applied in other fields of research to estimate complex models for data sets with missing observations. Examples of likelihood based methods are multilevel models. Multilevel methods are also called random effects, mixed, or hierarchical models.

Two advantages for using these models are that the dependency between measurements at successive time points is maintained, and that subjects with incomplete data are not excluded from the analysis. This means that, if a patient is missing one or more observations, the remaining available data from the other observations for that particular patient are used in the analysis [11]. When missing depends on the observed data, for example on previous HRQL outcome, the estimates provided by estimation procedures such as those of maximum likelihood used in the multi-level analysis, are unbiased [12]. Therefore, models like this are preferable because they incorporate all available information in the data and are less vulnerable to bias. This in contrast to an analysis confined to the complete cases [13]. Until recently, these modeling procedures were not available in most standard software packages used by the majority of clinical researchers. Some frequently used software programs of today offer this option. However, to our knowledge in the field of lung transplantation and HRQL no studies have been published comparing results obtained with one of these programs to results obtained with the commonly used software packages.

In the present study, we compared results obtained with three different methods of analysis: cross-sectional analysis, repeated measures ANOVA on complete cases, and multi-level analysis. We used the dimension 'energy' of the Nottingham Health Profile (NHP) with a maximum follow-up of almost 10 years after lung transplantation. This dataset was suitable for the present purpose, because it covered a long period of follow-up, it included different types of missing data, and depending on the period of follow-up, there was a rather substantial amount of missing assessments.

## Patients and Methods

### Patient population and HRQL measure

After lung transplantation patients were asked to fill in HRQL-questionnaires at one, four, seven, and subsequently every six months. The questionnaires consisted of a combination of generic, disease-specific, and domain-specific health status measures, including the Nottingham Health Profile (NHP) [14].

The NHP is a generic measure of health status designed to measure perceived health on six specific domains of life. For illustrative purposes, one outcome measure is considered in this study: the dimension energy of the NHP. NHP-energy scores are shown in the present study because they depict an important dimension of HRQL in LgTX patients. Possible scores range from 0 to 100. When interpreting the results, please note that higher scores represent lower experienced energy levels. Between November 1990 and September 2003, 239 patients filled in one or more HRQL questionnaires after transplantation, and were analyzed in the present study. The maximum period of follow-up was 109 months after transplantation.

### Data set

The numbers of completed and missing questionnaires were registered at all time points. For convenience of comparison, numbers of completed and missing questionnaires at 1, 13, 37, 73, and 109 months are shown in table 1. In our data set, three reasons for missingness can be distinguished. First incidental dropout, which means that a person has one or more missing forms in-between a series of completed forms. Secondly, dropout due to censoring, which includes patients that could not complete the questionnaire because their time since transplantation was shorter than that specific period of follow-up. For example, 20 patients did not complete the 13-month questionnaire, because they were transplanted less than 13 months before the moment we analyzed the data set. The last column shows the number of patients that died before a specific time point. For example 48 patients did not complete a questionnaire at 13 months after transplantation, because they had died within 13 months after transplantation.

**Table 1:** Numbers of completed and missing questionnaires

Time after transplantation	Completed questionnaires	Missing questionnaires		
		
months	number	Incidental number	Censored number	Deceased number
1	133	106	-	-
.				
13	115	56	20	48
.				
37	74	28	72	65
.				
73	45	15	103	76
.				
109	14	8	127	90

Patients: n = 239

### Methods of analyses

By means of a logistic regression model [15] we tested which type of missing occurred in our data. The analysis suggested that the probability a questionnaire was missing was dependent on previous HRQL measurements. Consequently, the use of a likelihood based method was appropriate. For further reading on the subject of testing for different types of missingness we refer to Hedeker and Gibbons [16].

*Cross-sectional analyses *were performed using descriptive statistics, including mean scores and standard errors, on all available cases at each time point. For these analyses, the SPSS program was used (SPSS 11.0; SPSS, Inc; Chicago). *Repeated measures on complete cases *were also performed in SPSS, using repeated measures analysis of variance including only those patients who had complete follow-up until 73 months after transplantation.

For the *multi-level analysis *the MLwiN software package for fitting multi-level models was used (version 1.10; Centre for Multilevel Modelling, Institute of Education, University of London, UK). In an additional analysis, the same results were obtained by using the mixed models option in SPSS (SPSS 12.0; SPSS, Inc; Chicago). For further reading on different software packages see Singer and Willet [17]. An SPSS syntax file is available from the authors on request.

In the modeling process, variables were included in the model sequentially. After each step, the goodness of fit was determined by the difference in deviance (-2*loglikelihood) between the present and the previous model, and the number of additional included variables compared to the previous model. We used the unconditional means model [17] as a starting point. Instead of describing change in the outcome over time, this model simply describes and partitions the outcome variation across patients [17]. Subsequently, time was added to the model (unconditional growth model [17]) based on the observed pattern of results of the cross sectional analysis.

Finally, a number of confounding variables was identified because of their expected influence on experienced energy after transplantation, based on the available literature. Demographic data like gender, age, and diagnosis could be of influence [18,19]. Diagnosis was categorized into 4 categories: 'alpha 1 antitrypsin deficiency', 'cystic fibrosis', 'emphysema' and 'other'. Furthermore, time spent on the waiting list, and the presence or absence of Bronchiolitis Obliterans Syndrome (BOS) which is characterized by a slowly progressive decline in lung function and is also associated with increased morbidity [2,20] were possible predictors. The severety of BOS was not taken into account. Presence of BOS was assessed according to the criteria of the International Society for Heart and Lung Transplantation [21], either on functional data, if there was sustained and significant decline in the forced expiratory volume in 1 second to less than 80% of a previously established baseline value, or on the presence of obliterative bronchiolitis in biopsies, even if the lung function had not deteriorated [2].

Finally, the calendar year in which a patient was transplanted was a possible predictor of NHP-energy scores after LgTX. After the 'unconditional growth model'[17] was built, an advanced model was fitted based on these possible predictors.

## Results

### Indication of the missing data problem and demographic characteristics

Table 1 shows the magnitude of the missing data problem. One month after transplantation 133 patients completed a HRQL questionnaire. At the end of the follow-up period, approximately 9 years after transplantation (109 months), 14 patients completed a questionnaire, 8 patients had an 'incidental-missing', 127 did not complete the questionnaire because their time since transplantation was shorter than 109 months (censoring), and 90 patients had died.

In table 2, the demographic characteristics of the patients in the study population are depicted.

**Table 2:** Characteristics of transplanted patients (n = 239)

Gender, Male n(%)	128 (53.6)
Age years, mean (range)	44 (20–64)
Diagnosis, n (%)	
alpha1 antitrypsin deficiency	59 (24.7)
Emphysema	41 (17.2)
Cystic fibrosis	48 (20.1)
Miscellaneous	91 (38.0)
Days on waiting list, mean (range)	465 (1–2207)
Patients with BOS, n (%)	67 (28.1)

Two hundred thirty nine patients were included. Mean age of this population was 44 years, and 53.6% were male. In our sample, the main diagnosis before lung transplantation was alpha 1 antitrypsin deficiency. Furthermore, 67 patients developed BOS at some time point after transplantation.

### NHP-energy scores

**Figure 1:**
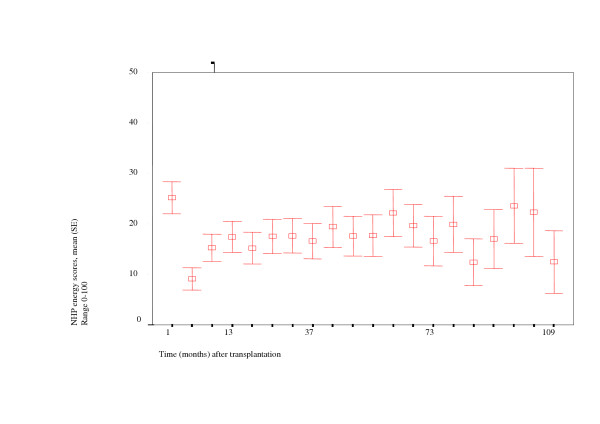
Results of cross sectional analysis

Results of *cross-sectional *analyses (mean and standard error per time point) are depicted graphically in figure 1. At each time point the analysis is based on a different group of patients, and consequently no changes over time could be assessed. One month after transplantation, mean NHP-energy scores are approximately 25 (range: 0–100), whereas the reference value for the general population is below 15. Four months after transplantation, means scores are below 10 (range: 0–100), and after that mean scores are around 15 (ranges 0–100 and 0–63 at all time points till 103 months and 109 months respectively), and remain more or less stable and within the reference value at the different points in time (in the different subgroups). Towards the end of the follow-up period mean scores seem to fluctuate. However, number of patients in these subgroups are relatively small, and results should be carefully interpreted.

**Figure 2:**
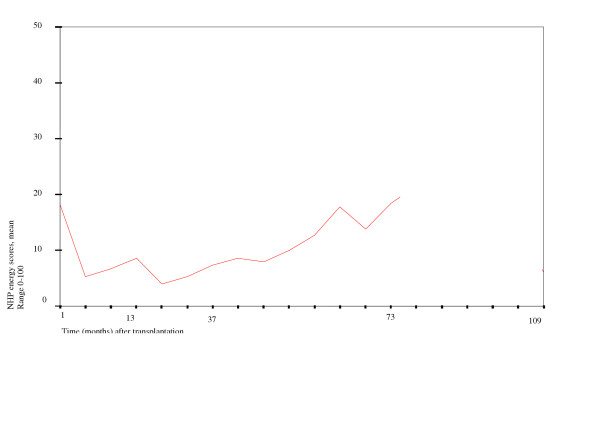
Results of repeated measures ANOVA on complete cases

To maintain a reasonable sample to analyze in the *repeated measures ANOVA on complete cases *we used a follow-up period of 73 months. This allowed for the inclusion of 19 patients in the analysis (figure 2). One month after transplantation, mean NHP-energy scores were just below 20. Between four and approximately 40 months mean scores are between 5 and 10, and after that scores increase, indicating worse health. Changes over time appeared to be not significant in this group and over this period.

Table 3 shows the three significant models, estimated with the *multi-level analysis*. The modeling procedure started with an unconditional means model, using of a constant term only. This constant has one fixed and two random parts. The fixed part can be interpreted as the mean score over all patients and time points (in this model approximately 19 points), whereas the random parts represent the variability within and between patients (not shown).

**Table 3:** Variables in various stages of the model

**Explanatory variables**	**Unconditional means model Estimate (SE)**	**Unconditional growth model Estimate (SE)**	**Final model Estimate (SE)**
***Fixed***			
Constant	19.40 (1.92)	18.16(2.42)	22.66 (3.05)
Time		-5.32 (3.76)	-10.71 (3.04)
Time square		4.54 (1.84)	5.69 (1.81)
Time third degree		-0.80 (0.34)	-0.92 (0.33)
Time forth degree		0.04 (0.02)	0.05 (0.02)
Age			0.56 (0.17)
BOS			23.73 (2.84)
Gender (male)			-8.00 (3.58)
			
-2*loglikelihood (IGLS)	12935.69	12778.50	12698.65

All effects significant, except for time in the unconditional growth model

**Figure 3:**
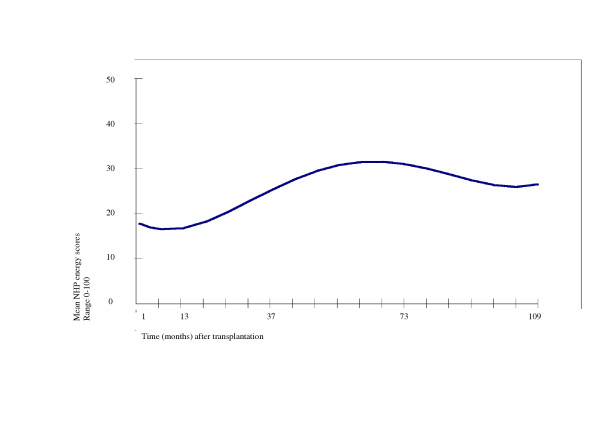
Estimated NHP-energy scores (unconditional growth model)

The unconditional means model was extended by including the time variable, and subsequently time square, time to the third degree, and time to the fourth degree, resulting in the unconditional growth model (figure 3). NHP energy scores that are estimated by the model can be compared to the results from cross-sectional and repeated measures ANOVA on complete cases.

After having estimated the changes over time, we added possible predictors to the model. First of all the presence of Bronchiolitis Obliterans Syndrome (BOS) was added. It was found that BOS had a statistically significant effect. Diagnosis did not contribute significantly to the model. Furthermore, neither time patients spent on the waiting list, nor calendar year of transplantation, nor the interaction between calendar year and time since transplantation contributed significantly. Age and gender however, provided a significant contribution to the model.

**Figure 4:**
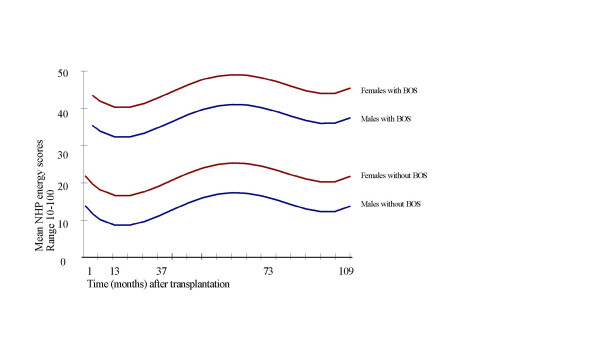
Estimated NHP energy scores (final model)

In figure 4 the predictions based on the estimates obtained from the final model are graphically displayed. The lines show mean NHP energy scores over time in transplanted males and females with and without BOS. Age was centered at 44 years (the mean age in our population) so that the lines correspond to 44-year-old subjects. With each year of age, estimated energy scores increased with 0.56 points (table 3), indicating that the experienced energy level declines when patients get older. After the development of BOS, the estimated energy scores increased with 23.73 points (table 3), and overall, male patients had an eight points lower energy score than females. Note that higher scores represent less perceived energy.

### Comparison of the different methods

**Figure 5:**
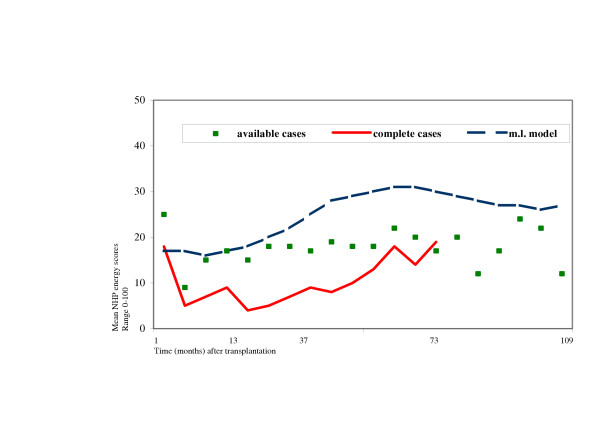
Comparison of available case, repeated measures ANOVA on complete cases, and multi-level analysis

Figure 5 displays the differences between the results estimated with the three methods of analysis. Patterns over time were comparable. However, clear differences were found concerning the mean scores, the number of included patients, and the period of follow-up.

*Cross-sectional analysis *of available cases showed mean scores that were more or less in-between the mean scores estimated with the other two methods. Furthermore, with this method, all patients were included, and results were analyzed until the maximum period of follow-up, 109 months after transplantation. However, no changes over time could be assessed.

*Repeated measures ANOVA on complete cases *showed the lowest scores compared to the other two methods, indicating better health. In this type of analysis, the smallest number of patients could be included, and results were analyzed until 73 months after transplantation, which was the shortest period of follow-up. Changes over time could be assessed.

*Multilevel analysis *showed higher predicted scores compared to the other two methods, indicating worse health. All patients and measurements were included in the analysis, and results were analyzed up to the maximum period of follow-up. Furthermore, changes over time could be assessed, and this method accounts for dependency between different measurements within a patient. In addition, predictors could be added to the model.

## Discussion

Missing data is a common problem in HRQL research. However, only few studies assessing HRQL in lung transplantation patients [1,8] openly addressed the problems associated with missing data: possible bias and loss of efficiency. In the present study, we compared the results of three different methods in a data set where depending on the period of follow-up, there was a substantial proportion of patients that did not complete all questionnaires. Methods were: cross sectional analyses, repeated measures analysis ANOVA on complete cases, and multi-level analysis. The estimated NHP energy scores were used to illustrate differences in results. Analyses showed that in our dataset patients with missing data differed from patients who completed all questionnaires, which means that patients who completed all questionnaires were not representative for the entire population of transplanted patients. Results showed that mean scores on NHP-energy were less favorable when estimated with cross-sectional analysis compared to the repeated measures ANOVA on complete cases.

The unconditional growth model estimated in the multi-level analysis, showed the least favorable energy scores compared to the other two methods. Patterns over time were comparable in all three methods.

The finding that scores estimated with the multi-level method were higher and thus less favorable compared to the complete case, and especially the cross sectional results, may raise questions. This can be explained by the fact that in the multi-level analysis, contrary to the other two methods, patients who have a missing questionnaire at a certain time point are not excluded from the analysis. The model estimates the subjects trend across time on the basis of whatever data that subject has, augmented by the time trend that is estimated for the sample as a whole, and effects of all covariates in the model [16].

Thus, in the multi-level model, scores on previous time points are taken into account in the estimation procedure, whereas in the cross sectional analysis the means are solely based on the observed scores at that point in time. Patients who drop out due to their worse health most likely have less favorable scores on previous time points. Complete exclusion of these patients from the analysis (repeated measures ANOVA) will lead to a lower, more favorable estimation of mean scores compared to the situation were estimations are based on worsening previous scores (multi-level analysis).

In addition, the fact that mean predicted scores were less favorable with the multi-level method compared to the other two methods indicates a reduction of bias. Both cross sectional and longitudinal means are based on results from patients who had better health states. Therefore, in the repeated measures ANOVA on complete cases, the selection of surviving patients that are capable to complete each questionnaire could also explain the lower, more favorable scores.

We have demonstrated with this study that, when analyzing a data set in which missing assessments occur, differences between results obtained with the various methods of analysis do exist. Depending on the research aim each of the three methods has its merits.

Cross sectional analysis are appropriate when health states at separate time points are under study rather than changes over time. When changes over time are relevant longitudinal analysis are preferred [9]. However, exclusion of patients with one or more missing data, which occurs when repeated measures analysis is used, results in conclusions based on, and only applicable to the particular subgroup of patients. This approach, however, may be legitimate or even necessary in order to confine the analysis on a specific subgroup, like surviving patients, who were able to complete all questionnaires. When the focus is on changes over time, multi-level analysis provides a good alternative to repeated measures ANOVA because with this method all available data are used in the analysis. This method gives unbiased estimates for most types of missing data, and, like repeated measures ANOVA, takes into account the dependency between different measurements within a patient. Finally, multi-level analysis proved to be very useful to analyze longitudinal changes, to include all available assessments, to reduce bias, and to include predictors.

When interpreting results from longitudinal studies on HRQL after lung transplantation, it is wise to be informed about the amount and type of missing data, the type of analysis which was performed, and the subgroup of patients the analysis was confined to. All these aspects determine the population and the circumstances, for example surviving patients without major complications, for which the results and conclusions described in the study are valid.

Because in the multi-level analysis all available assessments are used in the analysis, no reduction of power takes place. A result of this more efficient use of data is that predictors can be included in the model. This is in contrast to the repeated measures ANOVA, where due to the selection of patients with complete data, the power is reduced dramatically, and inclusion of predictors is impossible.

In conclusion, when longitudinal changes are under study, and missing data occur in the data set, Multilevel analysis is preferred to cross sectional and complete case analysis.

## Declaration of competing interests

The author(s) declare that they have no competing interests.

## Authors' contributions

KV was involved in acquisition of the HRQL data, carried out the statistical analysis and interpretation of the data, and drafted and revised the manuscript.

WP contributed to the conception and design of the study, supported carrying out the statistical analysis, supervised the analysis and critically revised the manuscript.

MS intellectually supported the research, and critically revised the manuscript.

WB was involved in acquisition and interpretation of the clinical data and critically revised the manuscript.

GK supervised the research and analysis and critically revised the manuscript

ETV supervised acquisition of the HRQL data, contributed to conception and design of the study, and critically revised the manuscript.

All authors read and approved the final manuscript.
